# Effects of elevated root zone CO_2_ on xerophytic shrubs in re-vegetated sandy dunes at smaller spatial and temporal scales

**DOI:** 10.1186/s40064-015-1091-7

**Published:** 2015-06-27

**Authors:** Huang Lei, Zhang Zhishan

**Affiliations:** Shapotou Desert Research and Experimental Station, Cold and Arid Regions Environmental and Engineering Research Institute, CAS, Lanzhou, 730000 China

**Keywords:** Elevated root zone CO_2_, Closed-air CO_2_ enrichment (CACE) system, Xerophytic shrubs, Re-vegetated sandy dunes

## Abstract

The below-ground CO_2_ concentration in some crusted soils or flooded fields is usually ten or hundred times larger than the normal levels. Recently, a large number of studies have focused on elevated CO_2_ in the atmosphere; however, only few have examined the influence of elevated root zone CO_2_ on plant growth and vegetation succession. In the present study, a closed-air CO_2_ enrichment (CACE) system was designed to simulate elevated CO_2_ concentrations in the root zones. The physio-ecological characteristics of two typical xerophytic shrubs *C*. *korshinskii* and *A*. *ordosica* in re-vegetated desert areas were investigated at different soil CO_2_ concentrations from March 2011 to October 2013. Results showed that plant growth, phenophase, photosynthetic rate, stomatal conductance, transpiration rate, and water use efficiency for the two xerophytic shrubs were all increased at first and then decreased with increasing soil CO_2_ concentrations, and the optimal soil CO_2_ concentration thresholds for *C*. *korshinskii* and *A*. *ordosica* were 0.554 and 0.317%, respectively. And *A. ordosica* was more tolerate to root zone CO_2_ variation when compared with *C. korshinskii*, possible reasons and vegetation succession were also discussed.

**Electronic supplementary material:**

The online version of this article (doi:10.1186/s40064-015-1091-7) contains supplementary material, which is available to authorized users.

## Background

The below-ground CO_2_ efflux from root and microbial respiration, the decomposition of various carbon pools as well as some improper soil management methods, such as compacted soil, over-irrigation, or flooding in agriculture land would lead to a relatively enriched CO_2_ environment in the rhizosphere (Huang et al. 1997; Boru et al. 2003), CO_2_ concentration in well-aerated soil is generally 1,000–2,000 μmol/mol (0.1–0.2%). By contrast, CO_2_ concentration in poorly aerated soils could reach up to 5–10% or sometimes even higher (Kammann 2001), which was almost 100-fold higher than atmospheric CO_2_ concentrations (De Jong and Schappert 1972; Norstadt and Porter 1984; He et al. 2010). This elevated root zone CO_2_ has shown influences on several key physiological processes of plants (He et al. 2004, 2007). It has been reported to enhance plant growth (Viktor and Cramer 2003; Cramer et al. 2005) although negative consequences for plant growth resulting from elevated root zone CO_2_ have also been reported (Cramer 2002), especially in combination with O_2_ deficiency (Boru et al. 2003). These contrary results could mainly due to the fact that the effects of elevated root zone CO_2_ on plant growth depended on a wide range of circumstances including plant species, soil pH, abiotic stress, the root zone CO_2_ concentration and so on (Boru et al. 2003; He et al. 2010). Such as in Stolwijk and Thimann (1957) showed that a 2% concentration of CO_2_ inhibited growth of pea (*Pisum sativum* L.) roots by 80%, whereas 6.5% CO_2_ did not affect root growth of oat (*Avena sative* L.) or barley (*Hordeum vulgare* L.).

Most of the fundamental understanding of plant responses to elevated CO_2_ comes from experiments involving greenhouses, open-top chambers, and free-air CO_2_ enrichment (FACE) systems (Ainsworth and Rogers 2007; Phillips et al. 2012). FACE experiments provide perhaps the best estimate of how plants and ecosystems will respond to high CO_2_ levels in the future (Long et al. 2004; Leakey et al. 2009). However, applying these exposure techniques in plant root zones is impossible. Recently, the aeroponic culture experiment system has been applied in plant root zones to elevated CO_2_ concentrations. The roots of each plantlet were inserted through holes, and the plant root zones were continuously aerated with gas stream (Boru et al. 2003; He et al. 2010). However, such experiments were mainly based on laboratory studies and were conducted for only short periods of time. Thus, a suitable device and the long-term monitoring of the effect of elevated root zone CO_2_ on xerophytic shrubs under natural conditions is lacking.

In sandy deserts areas such as the Tengger Desert, local moss crusts and algae crusts display very good formation and development following the sand dune stabilization (Li et al. 2004). And the crusted soils serving as a compact protection film that prevents the emission of soil CO_2_ into the atmosphere (Huang et al. 2014), which lead to an increased CO_2_ concentration in the root zones, and in turn, would have impact on the plant growth and vegetation pattern formation. On the other hand, CO_2_ is the primary substrate for photosynthesis in the green plants (Sun et al. 2011). Thus, how does this part of accumulated CO_2_ influence on the plant, known as the “fertilization effect” (Poorter 1993), or hampers the plant growth and production (Hank et al. 2006), relative few studies have examined this trade-off. It was hypothesized that each plant has its own optimal soil CO_2_ concentration thresholds. To support this hypothesis, in the present study, a closed-air CO_2_ enrichment (CACE) system based on gas permeation and diffusion properties was firstly designed to simulate elevated CO_2_ concentrations in plant root zones. And then the physio-ecological characteristics of two typical xerophytic shrubs in re-vegetated desert areas, *Caragana korshinskii* and *Artemisia ordosica* were investigated at gradient CO_2_ concentrations, finally, plant succession, re-vegetation, and ecosystem management in sandy areas were also discussed.

## Results

### Actual CO_2_ concentrations in soil

A total of 48 soil gas samples were taken every month during the 3-year study period. In the control group, the average CO_2_ concentrations in the root zones of *C. korshinskii* and *A. ordosica* were 659.504 ± 246.257 and 637.271 ± 334.098 μmol/mol, respectively. And as shown in Table 1, the elevated CO_2_ concentrations in the CACE system of 700, 1,000, 2,000, 5,000, 10,000, 20,000, and 50,000 μmol/mol resulted in 0.069, 0.086, 0.152, 0.317, 0.554, 0.626, and 0.797% increases in the soil profile. The actural CO_2_ concentration in the soil profile increased sharply with the increasing CO_2_ gradient from 700 to 10,000 μmol/mol, but when the CO_2_ concentration in the cylinders was large than 10,000 μmol/mol, the increasing rate of actural CO_2_ concentration in the soil profile was not obvious, and finally the soil CO_2_ concentrations would saturated. In addition, the magnitude of fluctuation decreased with the increasing soil CO_2_ concentrations, which also refer to the fluctuant soil CO_2_ concentrations in the soil profile.

**Table 1:** CO_2_ concentration in the steel cylinder and the actual CO_2_ concentration in the soil (μmol/mol)

CO_2_ concentration in the steel cylinder	700	1,000	2,000	5,000	10,000	20,000	50,000
Actual CO_2_ concentration in the soil (mean ± SE)	685 ± 430.891	856 ± 316.562	1,524 ± 304.371	3,172 ± 622.621	5,538 ± 693.676	6,260 ± 724.741	7,974 ± 894.483

### Pn, Tr, Gs, and WUE under different CO_2_ concentrations

**Figure 1:**
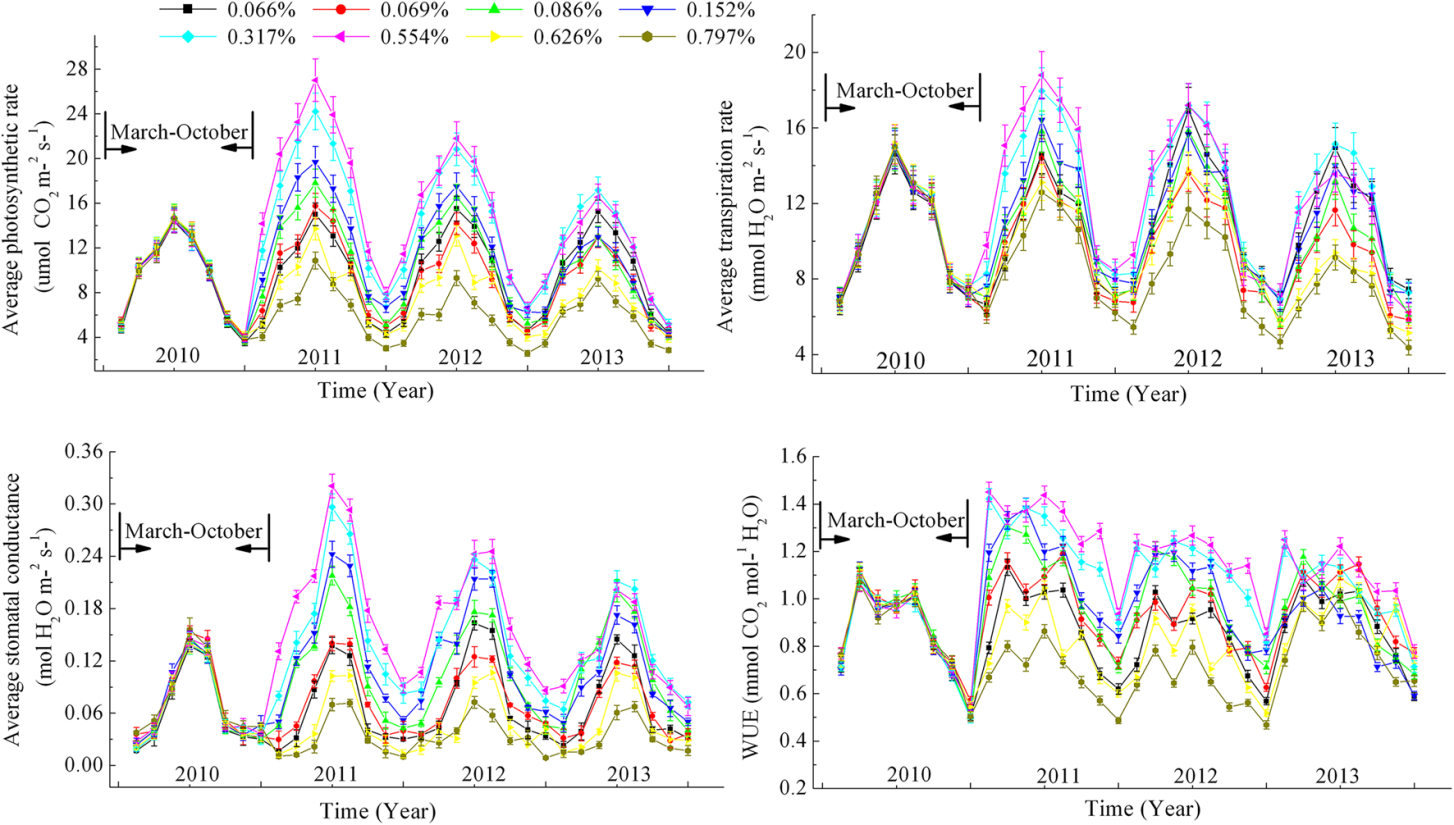
The average Pn, Tr, Gs and WUE of *C. korshinskii* during the experimental period (2010–2013).

**Figure 2:**
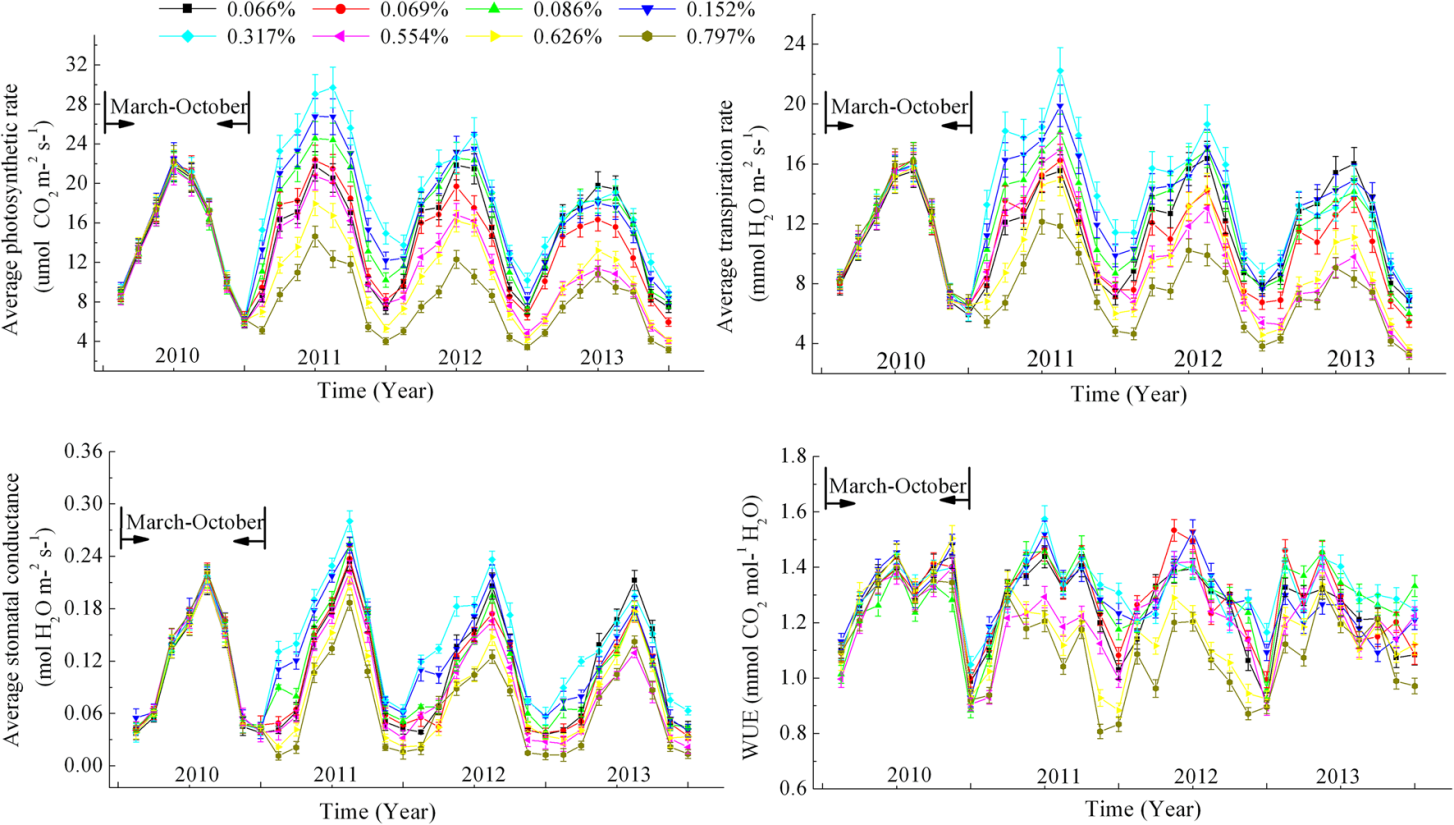
The average Pn, Tr, Gs and WUE of *A. ordosica* during the experimental period (2010–2013).

The relief valves of the CACE system were closed in 2010, as shown in Figures 1 and 2, the average Pn, Gs and Tr of the *C. korshinskii* and *A. ordosica* during the growing season (March 2010 to October 2010) had distinct seasonal variations with the single-peak curve and the highest occurred at June. But for WUE, the curve exhibited with a double-peak appearance, *C. korshinskii* reached its maximum in April and August and the WUE of *A. ordosica* reached its maximum in June and September. However, as shown in Additional file 1: Tables S1–S8, during the closed period of CACE system in 2010, the above measured four physio-ecological parameters of *C. korshinskii* and *A. ordosica* at different pots have showed no significant difference (*P* > 0.05). However, when the relief valves turn on from January 2011, the elevated root zone CO_2_ affected the Pn, Tr, Gs, and WUE of *C. korshinskii* and *A. ordosica* greatly, and there have showed a significant difference between different soil CO_2_ concentrations as seen in Additional file 1: Tables S1–S8 (*P* < 0.05). In short time scales, such as in 2011, the Pn, Tr, Gs, and WUE of *C. korshinskii* and *A. ordosica* were all increased with evaluated root zone CO_2_ concentrations under the threshold soil CO_2_ concentration (0.554% for *C. korshinskii* and 0.317% for *A. ordosica*). But above the threshold, the values of each measured parameters were smaller than those of the control groups which means that the high soil CO_2_ concentrations would inhibited the plants growth and development. On the other hand, in the 3 year time scale, evaluated root zone CO_2_ concentrations had a positive stimulation on plant growth, but after a period of adaptation, they would return to the normal level although the parameters were still larger than the control site, in addition, the difference between each CO_2_ concentration gradients at different years was not significant (*P* > 0.05) But below the threshold, the growth of plants represented by the above four measured parameters was all lower and they could not restored to normal levels. Specifically, for *C. korshinskii*, before the threshold optimal soil CO_2_ concentration (0.554%), each additional of 0.001% soil CO_2_ concentration, the Pn, Tr, Gs, and WUE was increased by 2.1–31.3%, 0.04–0.5%, 0.8–6.2%, 0.9–3.8%, respectively, but after exceeding this threshold, the Pn, Tr, Gs, and WUE was reduced by 5.4–15.4%, 0.07–0.2%, 0.8–6.2% and 0.2–0.7% for each additional of 0.001% soil CO_2_ concentration. And for *A. ordosica*, before the threshold optimal soil CO_2_ concentration (0.317%), each additional of 0.001% soil CO_2_ concentration would lead to the Pn, Tr, Gs, and WUE increase by 3.2–41.5%, 0.03–0.2%, 2.1–23.7%, 0.02–0.8%, respectively, but after exceeding this threshold, the Pn, Tr, Gs, and WUE was reduced by 0.2–1.2%, 0.006–0.07%, 0.09–0.5% and 0.01–0.06%.

### The phenophase of *C. korshinskii* and *A. ordosica* under different CO_2_ concentrations

**Figure 3:**
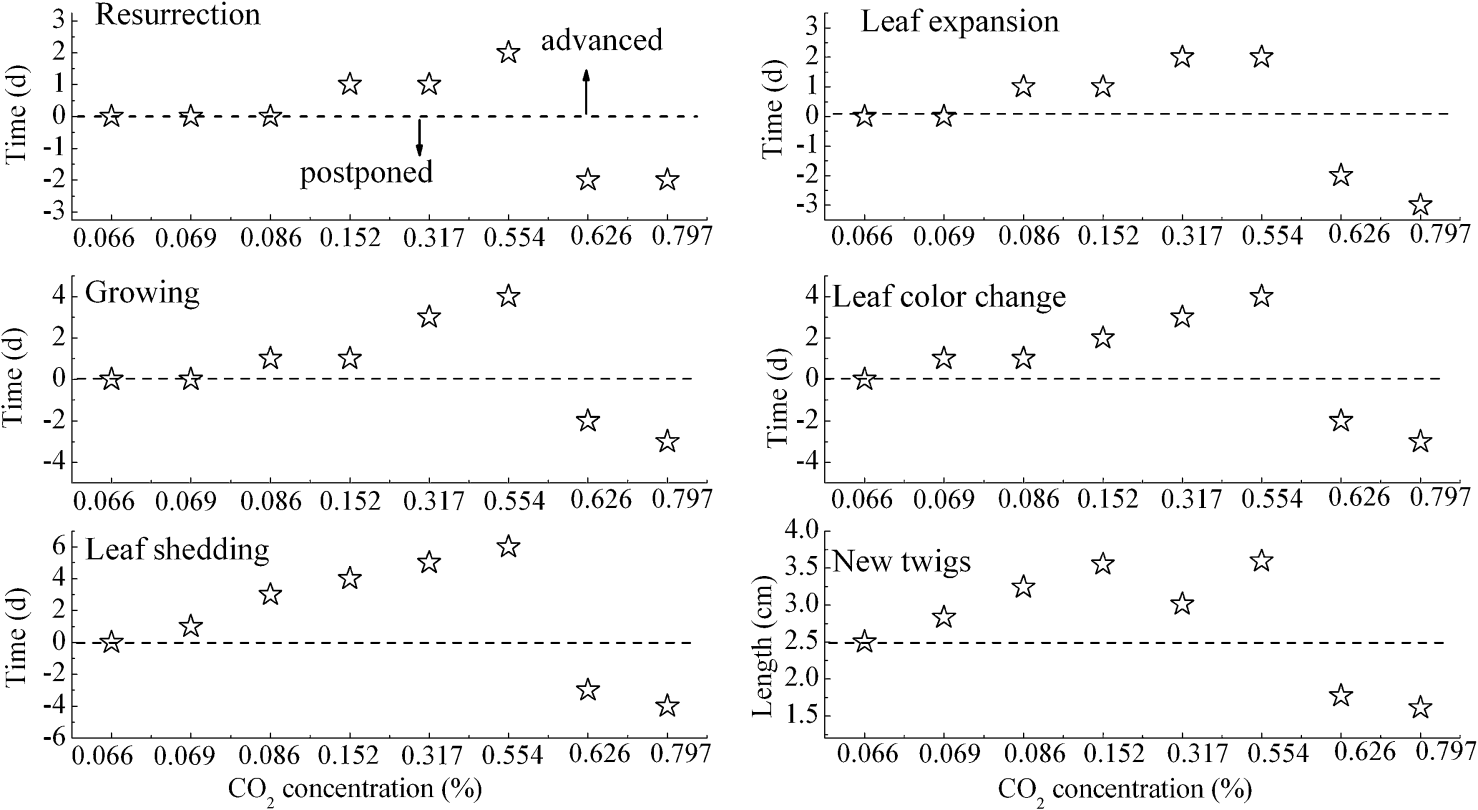
The difference of average phenodate every degree of soil CO_2_ concentration in *C. korshinskii* from March 2011 to October 2013 (the *vertical axis* of the graph represents the advanced or postponed days of plant phenology, which it was an integer value as 1, 2, 3 …, the *dotted line* was plant growing in the natural conditions, the upward means the plant phenology was advanced, and conversely the downwards means plant phenology was postponed. The *horizontal axis* represents the different CO_2_ concentration gradient).

**Figure 4:**
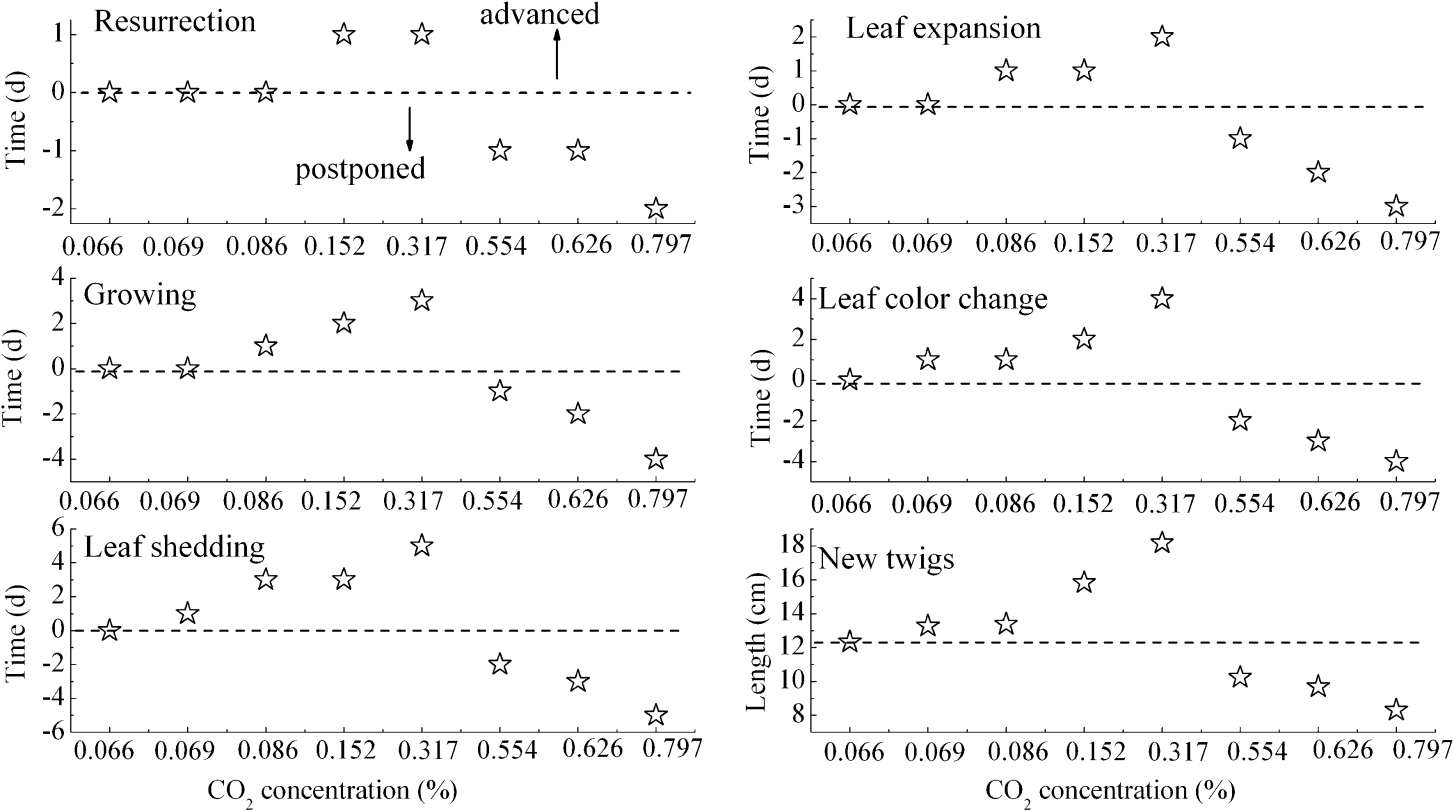
The difference of average phenodate every degree of soil CO_2_ concentration in *A. ordosica* from March 2011 to October 2013 (Ditto).

The elevated root zone CO_2_ affected the phenophase of *C. korshinskii* and *A. ordosica* significantly as seen in Figures 3 and 4. When the CO_2_ concentration in the soil was less than 0.554%, the phenological phase of *C. korshinskii*, including resurrection, leaf expansion, growing, leaf color change and leaf shedding stages was advanced with increasing soil CO_2_ concentrations, but when the CO_2_ concentration in the soil was larger than 0.554%, the elevated root zone CO_2_ would lead to a delayed phenophase comparing with the control groups. For *A. ordosica* as seen in Figure 4, this threshold of soil CO_2_ concentration was 0.317%. Same conclusion was also obtained from the new twigs of the both plants that the average annual length of the new branches of *C. korshinskii* and *A. ordosica* was 2.5 and 12.33 cm, and then reached its maximum 3.55 and 18.16 cm with increasing CO_2_ concentration until the threshold (0.554% for *C. korshinskii* and 0.317% for *A. ordosica*), but when exceeding the threshold value, they were lower than the normal plant growth and maintained at a relatively low level 1.61–1.77 cm and 8.31–9.69 cm, respectively. In addition, the phenophase for each treatment appeared almost simultaneously before the leaf color change stage. The phenological phase of the both plants were advanced or postponed for 1–4 days as the CO_2_ concentrations increased. However, a significant difference was observed specifically at the leaf shedding stages, and the phenological phase was advanced or postponed for 1–6 days.

## Discussion

Soil CO_2_ concentration in the rhizosphere is usually much larger than that in the near-surface atmosphere (Nepstad et al. 1994; Dan et al. 2014), particularly during rainy days. Soil CO_2_ production rates are generally 2–10 times higher in the wet season (December–May) than in the dry season (June–November) (Fierer et al. 2005). In this study, the CO_2_ concentrations under the *C. korshinskii* and *A. ordosica* communities could reach up to 6,086.9 and 4,746.2 μmol/mol, respectively, even with a small amount of rainfall (such as 4.8 mm in 26 June 2011) (Huang et al. 2014). However, high CO_2_ concentrations in the soil do not last long because of the gas diffusivity in the soil profile. CO_2_ concentrations return to a normal level with 4–5 days after rainfall (Huang et al. 2014). The photosynthetic, transpiration rate and stomatal conductivity were increased with increasing root zone CO_2_ under the optimal soil CO_2_ concentration thresholds for maximizing productivity. The reason was that there was more internal CO_2_ available to plants grown under elevated root zone CO_2_ as dissolved CO_2_ in the xylem sap could be carried upward in the stem when plants were transpiring. And the xylem transported CO_2_ could be then fixed in plant green tissues (He et al. 2010). As an important soil gas in the rhizosphere, CO_2_ has a significant influence on plant growth and development. It is a known source of inorganic carbon for photosynthesis (Bouma et al. 1997), and an increase in its concentration in soil results in a significant increase in the biomass of plants supplied with both NO_3_^−^ and NH_4_^+^ nutrition (Viktor and Cramer 2003). At the micro level, CO_2_ concentration plays a major role in determining the porosity, plasticity, and charge of cell membranes (Mitz 1979), which could thereby alter ion uptake and organic acid production (Yorgalevitch and Janes 1988). Such suite of mechanisms may well be responsible for increases in plant productivity, which means that the small increase of soil CO_2_ concentration in the rhizosphere would have positive effects on plant growth. But when root-zone CO_2_ was increased to 50%, a quarter of soybean (*Glycine max* L.) plants died (Boru et al. 2003). As high CO_2_-stressed plants cannot absorb sufficient moisture and nutrients from the soil, plant growth would be inhibited, inevitably leading to a depression of leaf photosynthesis, transpiration, yields, and production quality (Hank et al. 2006). Consequently, there should be a threshold of optimal CO_2_ concentration for different plants in the soil profile, and above which, the positive effect would transform to the negative ones. In addition, under the root zone CO_2_ enrichment, there may be the coexistence of the lamellar-structure and the disk-shaped-structure grana. And the accumulation of starch grains in the chloroplast of plant leaves under long-term root zone CO_2_ enrichment in the root zone could alter the arrangement of grana thylakoids, which maybe result in an inhibition of sun radiation absorption and a decline of plant photosynthesis (Sun et al. 2011). However, in the long term time scale, this may be a significant cause of the zonal distribution of plant formations in different region with different soil types.

In arid desert sandy areas, the crusted soils formed a compact protection film above the sand dunes, which have not only changed the water balance of the original soil-vegetation system, but also prevent soil CO_2_ emissions into the atmosphere and induced a high CO_2_ concentration around plant roots. Thus, based on the above results of the physio-ecological response to elevated root zone CO_2_ of the two typical xerophytic shrubs, we could deduce that aside from soil water, elevated root zone CO_2_ may be another key limiting factor that determines vegetation patterns in re-vegetated desert areas. As seen in Figures 1, 2, 3 and 4, for both vegetation types, plant growth or regeneration initially increased with increasing soil CO_2_ concentration, but started retreating after they reached its maximum in spite of continued soil CO_2_ concentration increasing. This conclusion has also verified by our previous results from long-term observation over 56 years of a sand-binding plantation in the Tengger desert that plant coverage increased from 10 to 35% 10 years after the re-establishment of vegetation, and then coverage gradually declined to 10% after 50 years (Li et al. 2004). CO_2_ production rates were strongly affected by the inter- and intra-annual variability in rainfall, especially in arid desert areas (Huang et al. 2014). As a constant rainfall not only causes soil CO_2_ accumulation but O_2_ deficiency in roots, which in turn, does plants severe damage. The difference of species response to CO_2_ concentration can cause changes in the relationship between interspecific competition. The WUE of *A. ordosica* was larger than *C. korshinskii*, and the difference of WUE of *A. ordosica* between different CO_2_ concentrations was more smoothly, which indicated that *A. ordosica* was more tolerate to drought stress and root zone CO_2_ variation when compared with *C. korshinskii,* so we may infer that the *A. ordosica* plant would be the dominant species in the vegetation succession. This hypothesis has also been verified by the recorded succession data and other results of sand-binding vegetation in the Shapotou area that *A. ordosica* may form a relatively stable climax community or plagioclimax, nearly 20 years later, and the originally planted shrubs such as *C. korshinskii and H. scoparium* will degrade and withdraw from the community (Li et al. 2004, 2013). The improvement of the soil surface in re-vegetated desert areas may elevate the root zone CO_2_ concentration and so eliminated planted xerophytic shrubs. Thus, based on the results of this study, we can conclude that in the early stage of vegetation restoration, increased soil CO_2_ concentrations through appropriate artificial methods such as planting grass patterns would be beneficial to plant growth, while minor human disturbances should be necessary in the latter to maintain the optimal CO_2_ concentrations, these suggestions are effective in ensuring the stability and efficient management of desert ecosystems in future vegetation reconstructions in sandy areas. Overall, we have demonstrated that each plant has its own appropriate CO_2_ concentrations, which could explain plant configuration or vegetation succession in re-vegetated sandy dunes. Although the ecological characteristics of two typical desert xerophytic shrubs were discussed in the present study, a mechanistic understanding of the physiological response of plants or the plant root system to elevated root zone CO_2_ still required further research. And precipitation, as the sole source of water replenishment in the desert areas (Li et al. 2004), its influence on vegetation succession when combined with elevated CO_2_ was still a challenge. Moreover, the CACE system employed in this study was simple and convenient for field operations, allowing for the easy simulation of the changes in other components (such as CH_4_ or N_2_O) of the soil air and the influence of such changes on the plants.

## Conclusions

Elevated root zone CO_2_ may have potential effects on plant growth and vegetation succession in the revegetated desert areas. In the present study, a closed-air CO_2_ enrichment (CACE) system was designed to simulate elevated CO_2_ concentrations in the root zones. The physio-ecological characteristics of two typical xerophytic shrubs, *C. korshinskii* and *A. ordosica* were studied. Results showed that plant growth, phenophase, photosynthetic rate, stomatal conductance, transpiration rate, and water use efficiency for the two xerophytic shrubs were all increased at first and then decreased with increasing soil CO_2_ concentrations, and the optimal soil CO_2_ concentration thresholds for *C. korshinskii* and *A. ordosica* were 0.554 and 0.317%, respectively. And *A. ordosica* was more tolerate to root zone CO_2_ variation when compared with *C. korshinskii*, which means that root zone CO_2_ concentration was one of the potential driving factors in plant succession in revegetated desert areas at smaller spatial and temporal scales. Furthermore, we predicted that grass planting patterns or minor human disturbances are effective in enhancing sustainability and management of desert ecosystems in future ecological constructions in sandy areas.

## Methods

### Study site

The study area is the Shapotou Desert Research and Experimental Station of the Chinese Academy of Sciences located in the Shapotou region at the southeastern margin of the Tengger Desert (37°32′N, 105°02′E). The climate at the site is characterized by abundant sunshine and low relative humidity. The average monthly relative humidity is a minimum of 33% in April and a maximum of 54.9% in August. The elevation of the area is 1,330 m, and the mean annual precipitation is 188.2 mm (according to meteorological records from 1956 to 2009), falling predominantly between June and September. The mean annual temperature is 9.6°C, and the mean monthly temperatures are −6.9°C in January and 24.3°C in July. The potential evapotranspiration during the growing season (May–September) is 2,300–2,500 mm. To protect the Baotou–Lanzhou railway line from sand burial, a non-irrigation vegetation protection system was established in 1965. After 50 years, the planted shrub vegetation was replaced gradually by dominant shrubs, such as *C. korshinskii*, *A. ordosica*, and *H. scoparium,* and by other herbaceous plants because of the decreasing water content in deeper soil layers (Li et al. 2004). Meanwhile, the propagation of numerous cryptogams on the fixed sand surface as well as the colonization of annual and perennial plant species promoted the succession and restoration of vegetation toward herb-dominated vegetation, which is similar to the primary vegetation types of the adjacent steppified desert and desert steppe (Li 2005).

### The closed-air CO_2_ enrichment (CACE) system

**Figure 5:**
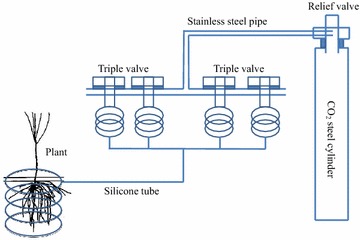
The closed-air CO_2_ enrichment (CACE) system.

Figure 5 shows that the CACE system is mainly composed of three key components: (1) the CO_2_ gas source: the steel gas cylinder was filled with prepared CO_2_ of different concentrations (i.e., 700, 1,000, 2,000, 5,000, 10,000, 20,000, and 50,000 μmol/mol in this study); (2) multi-channel design: triple valves were interconnected and then connected to the special steel cylinder with a relief valve; (3) closed-circuit design: silicone tubes were connected through the triple valves, and the outlet of the silicone tubes was sealed in the end. Given the good permeability of the silicone tube, the CO_2_ concentration around the silicone tube achieved equilibrium with the connected gas steel cylinders in a few hours. As the silicone tubes were wound spirally around the plant root, the CO_2_ concentration around the plant root system was evaluated. Besides, the gas extraction probes (Huang et al. 2014) were buried in the rhizosphere, and soil gas samples were collected monthly to measure the actual CO_2_ concentration around the plant root system. The CACE system was much cheaper than the FACE system and convenient in the simulation of below-ground elevated CO_2_ in natural conditions.

### Investigation methods

Field observation and sampling procedures were conducted from March 2010 to October 2013 in the Shapotou region. The *C. korshinskii* and *A. ordosica* plants were previously planted into 25-cm pots containing equal volumes of sand and meanwhile the silicone tubes in the CACE system were wound spirally around the plant root in March 2010, and the relief valves were closed until the start of the observations. There were totally 48 plants, including those in seven CO_2_ concentration gradients and the control groups were considered in the study, each of which had three replicates. After a year of growth the plant growth and physiological characters were observed from March 2011 to October 2013. The main honological phase, including the resurrection, leaf expansion, growing period, leaf color change, leaf shedding were carried out daily during the growth period on the southern side of the trees. Details of the phonological observation method have been described by Wan and Liu (1979). The resurrection is stage when leaf tip is visible at the end of bud, but before first leaf has unfolded to expose leaf petiole or leaf base. Leaf expansion is stage when its entire length has emerged from bud scale so its leaf stalk or petiole is visible at its point of attachment to stem, but before leaf has reached its full length or turn dark green or mature. For calculating the length of the growing season, we interpreted first leaf unfolding and leaf coloring as signs of start and end of the growing season (Guo et al. 2013). Leaf color change starts when the color of the leaves start to change from green into is light green, yellow, red, orange, or brown during late summer and autumn. Leaf coloring dates were recorded when 50% of leaves had changed color. Leaf shedding is the stage when more than half of the leafs are falling or has recently fallen from the plant, but not the one that has fallen due to disturbance or before it has reached the maturity. New twig growth were observed half a month during our experimental period, which determines the temporal accuracy of the dataset. Starting in 2010, three sunlit healthy leaves per replicate were randomly selected and labeled at the same position of the plants for repeated measurements, each leaf was measured three times, and the same leaves were measured in the following measurements. The photosynthetic rate (Pn), transpiration rate (Tr), and stomatal conductivity (Gs) were then subsequently measured monthly using the LI-6400 photosynthesis system (Li-Cor, Lincoln, NE). Each measurement began at a fixed time (10:00 am, Beijing local time). WUE was calculated as Pn/Tr (Hamid et al. 1990). Meanwhile, in order to get the actual CO_2_ concentrations in soil, the extracted soil air samples in each pot were brought monthly to the laboratory immediately for gas chromatography (GC, Agilent 6820, Agilent Technologies, USA).

### Data analysis

The plant photosynthetic rate (Pn), stomatal conductance (Gs), transpiration rate (Tr), and water use efficiency (WUE) data for *C. korshinskii* and *A. ordosica* at different CO_2_ concentration gradients were compared by single factor analysis of variance (One-way ANOVA), and Tukey’s test were used for post hoc multiple comparisons. These analyses were conducted using SPSS 13 package (SPSS 13.0 Inc., Chicago, USA). The graphic plotting was conducted with Origin 7.0 software (OriginLab Corporation, Northampton, MA, USA).

## Additional file

Additional file 1:
**Tables S1–S8.** Mean Pn, Tr, Gs, and WUE of* C. korshinskii* and *A. ordosica* under different CO_2_ concentration gradients during our experimental years (2010–2013).
