# Supplementary material for: Effects of elevated root zone CO2 on xerophytic shrubs in re-vegetated sandy dunes at smaller spatial and temporal scales
Source: Springerplus. 2015 Jun 27;4:299. doi: 10.1186/s40064-015-1091-7 (PMC4483174; doi:10.1186/s40064-015-1091-7)
Supplement: Additional file 1: — Tables S1–S8. Mean Pn, Tr, Gs, and WUE of C. korshinskii and A. ordosica under different CO2 concentration gradients during our experimental years (2010–2013). [file 40064_2015_1091_MOESM1_ESM.docx]

| CO_2_ | 0.066% | 0.069% | 0.086% | 0.152% | 0.317% | 0.554% | 0.626% | 0.797% |
| --- | --- | --- | --- | --- | --- | --- | --- | --- |
| 2010 | 8.982±1.415^Aa^ | 9.402±1.381^Aa^ | 9.316±1.455^Aa^ | 9.193±1.392^Aab^ | 9.285±1.439^Ab^ | 9.258±1.367^Ac^ | 9.464±1.411^Aa^ | 9.245±1.393^Aa^ |
| 2011 | 9.447±1.415^Ca^ | 10.239±1.431^Ba^ | 11.836±1.627^ABa^ | 13.409±1.774^Aa^ | 16.390±2.134^ABa^ | 18.502±2.339^Ba^ | 8.319±1.144^Ca^ | 6.487±0.941^Cab^ |
| 2012 | 9.951±1.476^Ba^ | 9.110±1.189^BCa^ | 11.030±1.482^ABa^ | 11.839±1.548^ABab^ | 14.336±1.837^Aab^ | 15.075±1.896^Aab^ | 8.000±1.055^BCa^ | 5.453±0.780^Cb^ |
| 2013 | 9.882±1.393^ABa^ | 8.498±1.121^ABCa^ | 8.591±1.111^ABCa^ | 8.874±1.158^ABCb^ | 11.752±1.527^Aab^ | 11.417±1.420^Abc^ | 6.723±0.768^BCa^ | 5.662±0.789^Cb^ |

Table S1 Mean photosynthetic rate of the *C. korshinskii* under different CO_2_ concentration gradients during our experimental years

Values represents means ± SE. Different capital letters indicate significant difference in the mean photosynthetic rate of the *C. korshinskii* at the same year among different CO_2_ concentration gradients; different small letters denote significant difference in the mean photosynthetic rate among different years in the same CO_2_ concentration (p < 0.05).

Table S2 Mean transpiration rate of the *C. korshinskii* under different CO_2_ concentration gradients during our experimental years

| CO_2_ | 0.066% | 0.069% | 0.086% | 0.152% | 0.317% | 0.554% | 0.626% | 0.797% |
| --- | --- | --- | --- | --- | --- | --- | --- | --- |
| 2010 | 10.213±1.045^Aa^ | 10.447±1.050^Aa^ | 10.450±1.032^Aa^ | 10.420±1.057^Aa^ | 10.612±1.051^Ab^ | 10.490±1.050^Ab^ | 10.648±1.073^Aa^ | 10.532±1.076^Aa^ |
| 2011 | 10.213±1.045^BCa^ | 10.047±1.047^BaC^ | 10.896±1.080^ABCa^ | 11.587±1.169^ABCa^ | 13.055±1.409^AaB^ | 13.950±1.474^Aa^ | 9.925±0.861^BCa^ | 9.163±0.906^Cab^ |
| 2012 | 11.680±1.237^Aa^ | 10.120±0.932^ABa^ | 11.102±1.086^ABa^ | 11.444±1.067^ABa^ | 12.656±1.293^A a^ | 12.754±1.321^Aa^ | 10.483±0.853^ABa^ | 8.397±0.878^Bab^ |
| 2013 | 10.603±1.059^ABa^ | 8.386±0.790 ^BCa^ | 9.070±0.876^ABCa^ | 10.193±0.997^ABa^ | 11.273±1.210^Ab^ | 10.426±1.075^ABb^ | 7.222±0.584^Cb^ | 6.703±0.631^Cb^ |

Values represents means ± SE. Different capital letters indicate significant difference in the mean transpiration rate of the *C. korshinskii* at the same year among different CO_2_ concentration gradients; different small letters denote significant difference in the mean transpiration rate among different years in the same CO_2_ concentration (p < 0.05).

Table S3 Mean stomatal conductivity of the *C. korshinskii* under different CO_2_ concentration gradients during our experimental years

| CO_2_ | 0.066% | 0.069% | 0.086% | 0.152% | 0.317% | 0.554% | 0.626% | 0.797% |
| --- | --- | --- | --- | --- | --- | --- | --- | --- |
| 2010 | 0.063±0.017^Aa^ | 0.073±0.018^Aa^ | 0.067±0.017^Aa^ | 0.071±0.017^Aa^ | 0.067±0.016^Ab^ | 0.072±0.017^Ac^ | 0.068±0.016^Aa^ | 0.076±0.016^Aa^ |
| 2011 | 0.063±0.017^DEa^ | 0.074±0.016^CDEa^ | 0.111±0.023^BCDa^ | 0.130±0.026^BCa^ | 0.161±0.029^ABa^ | 0.195±0.028^Aa^ | 0.044±0.013^Eb^ | 0.030±0.009^Eb^ |
| 2012 | 0.077±0.019^CDa^ | 0.076±0.012^CDa^ | 0.106±0.018^BCa^ | 0.127±0.022^ABa^ | 0.147±0.022^ABa^ | 0.166±0.021^Aab^ | 0.050±0.012^Db^ | 0.037±0.007^Db^ |
| 2013 | 0.067±0.017^BCDa^ | 0.063±0.013^CDa^ | 0.109±0.021^ABa^ | 0.097±0.017^ABCa^ | 0.125±0.019^Aab^ | 0.125±0.017^Abc^ | 0.047±0.013^Db^ | 0.031±0.007^Db^ |

Values represents means ± SE. Different capital letters indicate significant difference in the mean stomatal conductivity of the *C. korshinskii* at the same year among different CO_2_ concentration gradients; different small letters denote significant difference in the mean stomatal conductivity among different years in the same CO_2_ concentration (p < 0.05).

Table S4 Mean water use efficiency of the *C. korshinskii* under different CO_2_ concentration gradients during our experimental years

| CO_2_ | 0.066% | 0.069% | 0.086% | 0.152% | 0.317% | 0.554% | 0.626% | 0.797% |
| --- | --- | --- | --- | --- | --- | --- | --- | --- |
| 2010 | 0.845±0.072^Aa^ | 0.871±0.064^Aa^ | 0.856±0.073^Aa^ | 0.852±0.069^Ab^ | 0.841±0.071^Ac^ | 0.853±0.063^Ac^ | 0.861±0.068^Aab^ | 0.849±0.065^Aa^ |
| 2011 | 0.894±0.065^CDa^ | 0.994±0.057^BCa^ | 1.060±0.073^BCa^ | 1.135±0.070^ABa^ | 1.235±0.061^Aa^ | 1.304±0.058^Aa^ | 0.814±0.054^DEab^ | 0.687±0.043^Ebc^ |
| 2012 | 0.824±0.055^DEa^ | 0.881±0.050^CDEa^ | 0.971±0.063^BCDa^ | 1.012±0.062^ABCab^ | 1.110±0.050^ABab^ | 1.160±0.048^Aab^ | 0.744±0.052^EFb^ | 0.635±0.040^Fc^ |
| 2013 | 0.906±0.059^BCDa^ | 0.987±0.050^ABCa^ | 0.931±0.060^ABCDa^ | 0.856±0.056^CDb^ | 1.028±0.057^ABb^ | 1.078±0.052^Ab^ | 0.916±0.044^BCDa^ | 0.820±0.049^Dab^ |

Values represents means ± SE. Different capital letters indicate significant difference in the mean s water use efficiency of the *C. korshinskii* at the same year among different CO_2_ concentration gradients; different small letters denote significant difference in the mean water use efficiency among different years in the same CO_2_ concentration (p < 0.05).

Table S5 Mean photosynthetic rate of the *A. ordosica* under different CO_2_ concentration gradients during our experimental years

| CO_2_ | 0.066% | 0.069% | 0.086% | 0.152% | 0.317% | 0.554% | 0.626% | 0.797% |
| --- | --- | --- | --- | --- | --- | --- | --- | --- |
| 2010 | 14.216±2.047^Aa^ | 14.526±2.056^Aa^ | 13.948±2.029^Aa^ | 14.666±2.054^Ab^ | 14.602±2.018^Ab^ | 13.973±2.016^Aa^ | 14.523±2.039^Aa^ | 14.516±2.048^Aa^ |
| 2011 | 14.807±1.948^CDa^ | 15.835±1.972^BCDa^ | 18.250±2.088^ABCa^ | 20.211±2.071^ABa^ | 22.713±2.062^Aa^ | 14.377±1.822^CDEa^ | 11.715±1.628^DEab^ | 9.131±1.381^Eb^ |
| 2012 | 15.035±1.969^ABCa^ | 13.694±1.685^ABCa^ | 16.262±1.991^ABa^ | 17.053±1.946^Aab^ | 18.082±1.855^Aab^ | 11.551±1.504^BCDab^ | 10.564±1.515^CDab^ | 7.613±1.101^Db^ |
| 2013 | 14.594±1.699^Aa^ | 12.352±1.372^ABa^ | 14.431±1.511^Aa^ | 14.297±1.283^Ab^ | 15.349±1.247^Ab^ | 8.379±0.970^Cb^ | 8.933±1.172^BCb^ | 7.252±1.004^Cb^ |

Values represents means ± SE. Different capital letters indicate significant difference in the mean photosynthetic rate of the *A. ordosica* at the same year among different CO_2_ concentration gradients; different small letters denote significant difference in the mean photosynthetic rate among different years in the same CO_2_ concentration (p < 0.05).

Table S6 Mean transpiration rate of the *A. ordosica* under different CO_2_ concentration gradients during our experimental years

| CO_2_ | 0.066% | 0.069% | 0.086% | 0.152% | 0.317% | 0.554% | 0.626% | 0.797% |
| --- | --- | --- | --- | --- | --- | --- | --- | --- |
| 2010 | 10.771±1.297^Aa^ | 11.142±1.338^Aa^ | 11.247±1.298^Aa^ | 11.133±1.296^Ab^ | 11.138±1.299^Ab^ | 11.093±1.281^Aa^ | 11.046±1.271^Aa^ | 11.408±1.300^Aa^ |
| 2011 | 11.308±1.153^CDa^ | 11.915±1.169^BCa^ | 13.555±1.206^ABCa^ | 14.994±1.248A^Ba^ | 16.638±1.241^Aa^ | 12.234±1.221^BCa^ | 10.234±1.177^CDab^ | 8.384±1.002^Db^ |
| 2012 | 11.913±1.128^ABCa^ | 10.438±0.997^BCa^ | 12.623±1.215^ABa^ | 12.905±1.161^ABab^ | 14.021±1.236^Aab^ | 9.163±0.941^CDab^ | 9.404±1.183^CDab^ | 7.218±0.863^Db^ |
| 2013 | 11.764±1.228^Aa^ | 9.823±1.067^ABa^ | 10.766±1.093^Aa^ | 11.867±1.057^Aab^ | 11.549±0.907^Ab^ | 6.819±0.778^Cb^ | 7.505±0.958^BCb^ | 6.297±0.749^Cb^ |

Values represents means ± SE. Different capital letters indicate significant difference in the mean transpiration rate of the *A. ordosica* at the same year among different CO_2_ concentration gradients; different small letters denote significant difference in the mean transpiration rate among different years in the same CO_2_ concentration (p < 0.05).

Table S7 Mean stomatal conductivity of the *A. ordosica* under different CO_2_ concentration gradients during our experimental years

| CO_2_ | 0.066% | 0.069% | 0.086% | 0.152% | 0.317% | 0.554% | 0.626% | 0.797% |
| --- | --- | --- | --- | --- | --- | --- | --- | --- |
| 2010 | 0.104±0.024^A a^ | 0.110±0.025^Aa^ | 0.108±0.024^Aa^ | 0.113±0.024^Aa^ | 0.108±0.025^Aa^ | 0.110±0.024^A a^ | 0.106±0.024^Aa^ | 0.113±0.026^Aa^ |
| 2011 | 0.115±0.027^ABa^ | 0.119±0.026^ABa^ | 0.133±0.026^ABa^ | 0.149±0.024^Aa^ | 0.161±0.026^Aa^ | 0.107±0.026^ABa^ | 0.090±0.025^ABa^ | 0.076±0.024^Bb^ |
| 2012 | 0.103±0.023^ABCa^ | 0.096±0.020^ABCa^ | 0.102±0.019^ABCa^ | 0.126±0.018^ABa^ | 0.145±0.021^Aa^ | 0.089±0.018^BCa^ | 0.074±0.015^BCa^ | 0.065±0.016^Cb^ |
| 2013 | 0.108±0.024^ABa^ | 0.090±0.019^ABa^ | 0.094±0.016^ABa^ | 0.102±0.017^ABa^ | 0.124±0.016^Aa^ | 0.065±0.014^Bb^ | 0.081±0.020^ABa^ | 0.060±0.017^Bb^ |

Values represents means ± SE. Different capital letters indicate significant difference in the mean stomatal conductivity of the *A. ordosica* at the same year among different CO_2_ concentration gradients; different small letters denote significant difference in the mean stomatal conductivity among different years in the same CO_2_ concentration (p < 0.05).

Table S8 Mean water use efficiency of the *A. ordosica* under different CO_2_ concentration gradients during our experimental years

| CO_2_ | 0.066% | 0.069% | 0.086% | 0.152% | 0.317% | 0.554% | 0.626% | 0.797% |
| --- | --- | --- | --- | --- | --- | --- | --- | --- |
| 2010 | 1.291±0.059^Aa^ | 1.278±0.054^Aa^ | 1.207±0.061^Aa^ | 1.291±0.065^Aa^ | 1.285±0.049^Aa^ | 1.229±0.065^Aa^ | 1.288±0.069^Aa^ | 1.242±0.058^Aa^ |
| 2011 | 1.277±0.053^ABa^ | 1.301±0.050^Aa^ | 1.323±0.050^Aa^ | 1.332±0.038^Aa^ | 1.355±0.044^Aa^ | 1.150±0.044^BCa^ | 1.121±0.057^Cb^ | 1.060±0.065^Cb^ |
| 2012 | 1.228±0.060^ABa^ | 1.286±0.062^Aa^ | 1.261±0.050^Aa^ | 1.300±0.047^Aa^ | 1.279±0.034^Aa^ | 1.231±0.059^ABa^ | 1.104±0.045^BCab^ | 1.034±0.045^Cb^ |
| 2013 | 1.227±0.036^Ba^ | 1.257±0.050^ABa^ | 1.338±0.025^Aa^ | 1.206±0.025^BCa^ | 1.327±0.028^Aa^ | 1.227±0.033^Ba^ | 1.185±0.029^BCb^ | 1.129±0.042^Cab^ |

Values represents means ± SE. Different capital letters indicate significant difference in the mean s water use efficiency of the *A. ordosica* at the same year among different CO_2_ concentration gradients; different small letters denote significant difference in the mean water use efficiency among different years in the same CO_2_ concentration (p < 0.05).
